# Efficacy and Safety of Poly‐l‐Lactic Acid for Correction of Midfacial Volume Loss and Contour Defects: A Prospective, Multicenter, Randomized, Parallel‐Controlled, Evaluator‐Blinded, Superiority Trial

**DOI:** 10.1111/jocd.70230

**Published:** 2025-07-18

**Authors:** Yi Zhang, Xinling Zhang, Xuejie Gao, Yaqi Wei, Wenjiang Qian, Zhongyang Sun, Jinping Ding, Shiwei Bao, Rongxin Ren, Hongyi Zhao

**Affiliations:** ^1^ Department of Plastic Surgery Beijing Hospital, National Center of Gerontology, Institute of Geriatric Medicine, Chinese Academy of Medical Sciences Beijing China

**Keywords:** midfacial contour defects, midfacial volume loss, poly‐l‐lactic acid, superiority clinical trial

## Abstract

**Background:**

Poly‐l‐lactic acid (PLLA) is widely used in esthetic medicine due to its excellent biocompatibility and biodegradability. This study aimed to evaluate the effectiveness and safety of PLLA facial filler in correcting midfacial volume loss and/or contour defects.

**Methods:**

In this prospective, multicenter, randomized, assessor‐blinded, superiority clinical trial, 331 subjects were randomly assigned to receive either PLLA (experimental group) or hyaluronic acid (HA, control group). Efficacy was assessed using the Midfacial Volume Scale (MMVS) and Global Aesthetic Improvement Scale (GAIS). Safety was evaluated based on adverse events.

**Results:**

The PLLA group demonstrated significantly higher efficacy in MMVS scores compared to the HA group (90.57% vs. 51.01%, difference of 39.56%, 95% CI: 30.34%–48.78%). These results were consistent across worst‐case and per‐protocol analyses. Secondary outcomes revealed higher MMVS and GAIS scores in the PLLA group at 6 months (MMVS: 93.04% vs. 69.33%, GAIS investigator rating: 99.37% vs. 86.67%) and 12 months (MMVS: 84.91% vs. 46.98%, GAIS investigator rating: 94.34% vs. 74.50%) (*p* < 0.05). Participant satisfaction surveys showed higher satisfaction in the PLLA group (*p* < 0.05). Safety analysis revealed no significant differences in vital signs, laboratory tests, or adverse events (*p* > 0.05), with a slightly higher incidence of injection site reactions in the PLLA group, which resolved within 1–3 days.

**Conclusion:**

The comprehensive analysis of results indicates that PLLA is a safe and effective treatment for the correction of midfacial volume loss and midfacial contour defects.

## 1 Introduction

With the rapid development of the medical esthetics industry, nonsurgical esthetic procedures, especially injectable fillers, have garnered widespread attention. Midfacial filling is used to improve facial contours, restore volume, and reduce wrinkles. Commonly used filling materials include cross‐linked hyaluronic acid gels, collagen, and polymethylmethacrylate (PMMA). However, these materials have certain limitations in terms of degradation rate, tissue compatibility, and long‐term efficacy. Cross‐linked hyaluronic acid gels, while offering good filling effects, degrade quickly and require frequent injections, with potential toxicity risks from residual chemical cross‐linking agents after degradation [1, 2]. Collagen fillers, primarily derived from animal sources or recombinant human collagen, degrade quickly, and animal‐derived collagen may cause allergic reactions. These are mainly suitable for fine wrinkle filling, with limited effects on severe depressions and deep wrinkles [3]. PMMA fillers, composed mainly of hydroxyapatite, are commonly used for bone repair, but when used for soft tissue filling, they may provoke inflammatory reactions and the formation of tissue nodules [4, 5].

Poly‐l‐lactic acid (PLLA) has gained widespread application in tissue engineering and medical esthetics due to its excellent biocompatibility and degradability. PLLA slowly degrades in the body into lactic acid and carbon dioxide, being fully metabolized without residue, while stimulating collagen production to achieve lasting facial rejuvenation effects [6, 7]. Compared to traditional hyaluronic acid fillers, PLLA fillers degrade more slowly, with effects lasting 1–2 years. They have been widely used for facial wrinkles, tissue defects, and soft tissue remodeling, with proven clinical safety and efficacy. Furthermore, the early clinical applications of PLLA were primarily focused on the correction of HIV‐associated facial lipoatrophy. Studies have demonstrated that PLLA can significantly restore facial volume loss, enhance skin thickness, and improve the quality of life in HIV patients [8, 9]. With ongoing research, PLLA fillers have been successfully applied to correct soft tissue defects in various parts of the body, such as for acne scars, skin grafts, tissue atrophy posttrauma, irregular scars after rhinoplasty, glabellar wrinkles, nasolabial folds, and other soft tissue injuries [10, 11, 12]. However, there are issues in the preparation process of PLLA fillers, such as high cost, difficulty in purification, and limitations in biological safety. Additionally, commonly used suspending agents like sodium carboxymethyl cellulose (CMC) still have room for improvement in terms of tissue compatibility and biological activity [13]. Therefore, this study selects PLLA as a midfacial filler material to optimize its preparation process, improve biological safety, and further evaluate its clinical effectiveness and application value.

## 2 Methods

### 2.1 Study Design

This study is a prospective, multicenter, randomized, parallel‐controlled, assessor‐blinded, superiority clinical trial conducted across four centers: Peking Hospital, West China Hospital of Stomatology, First People's Hospital of Hangzhou, and the Skin Disease Hospital of the Chinese Academy of Medical Sciences.

#### 2.1.1 Inclusion Criteria

1. Age ≥ 18 years; 2. Assessor‐blinded evaluation based on the Medicis Midface Volume Scale (MIMVS) [14], with inclusion criteria requiring both sides of the midface MMVS scores to be between 2 and 4 points; 3. The subject must voluntarily participate in this trial and sign an informed consent form.

#### 2.1.2 Exclusion Criteria

1. Facial symmetry assessment showing moderate to severe abnormalities (MMVS score difference > 1 point); 2. Acute infection, active skin diseases (such as eczema, rosacea), scars or deformities, cancer or precancerous lesions, or prior local skin radiotherapy in the treatment area or surrounding areas; 3. Recent facial esthetic surgery injections, facelift surgery, or planned procedures, such as permanent fillers, semi‐permanent fillers, autologous fat transfer, or facial surgeries, or having received hyaluronic acid or collagen‐based fillers within 12 months before enrollment, neurotoxin treatment within 9 months, or laser, radiofrequency, or similar treatments within 6 months; 4. Dental or oral issues incompatible with inclusion criteria, or having undergone dental or sinus surgery within 6 months prior to enrollment; 5. Plan to significantly change body weight during the trial (BMI fluctuation ≥ 2), such as through exercise or medication‐induced weight loss.

Based on the type of injected product, participants were divided into the PLLA group (experimental group) and the HA group (control group). Before use, PLLA was reconstituted with normal saline to form a suspension. Specifically, 246 mg of PLLA was dissolved in 5 mL of saline, resulting in a final concentration of 49.2 mg/mL. In the PLLA treatment group, the average injection volume was 6.94 ± 2.54 mL on the left side of the face and 6.85 ± 2.64 mL on the right side. In the HA control group, the average injection volume was 1.57 ± 0.60 mL on the left side and 1.52 ± 0.58 mL on the right side. The difference between groups was statistically significant (*p* < 0.05). The difference in volume is due to the distinct composition and mechanism of action of PLLA and HA, with dosing based on product guidelines and clinical protocols.

Both products were administered using a sharp needle for bolus injection at the supraperiosteal level and a blunt cannula for fanning injection in the deep fat layer. Before injection, the product was gently swirled to achieve a uniform suspension and drawn into a sterile syringe using a 25G or larger needle. The injection was performed within 30 min. Superficial injections could cause blanching, in which case the needle was withdrawn, and the area was gently massaged. Each subject in the experimental group will receive a maximum of three injection treatments, with each treatment separated by a 4‐week interval. If the optimal midfacial filling is achieved or after three injections, treatment will be discontinued. Each subject in the control group will receive a maximum of two injection treatments, with each treatment separated by a 4‐week interval. If the optimal midfacial filling is achieved or after two injections, treatment will be discontinued. The subjects provided a signed informed consent form for participation.

### 2.2 Outcome Assessments

Both groups of subjects will receive product injections into the deep dermis, subcutaneous tissue, or periosteum, filling and increasing tissue volume to correct midfacial volume loss and/or midfacial contour defects. The efficacy will be assessed using the commonly used clinical scale for evaluating midfacial fullness, the Medicis Midface Volume Scale (MIMVS). A blinded assessor will conduct onsite evaluations to avoid subjective bias resulting from the researcher's knowledge of the group allocation. The primary endpoint will be the improvement in MIMVS scores at 12 months after the last injection compared to baseline. If, relative to baseline, both sides of the midface show a decrease in MIMVS score by one level or more, the treatment will be considered effective; otherwise, it will be deemed ineffective. Secondary endpoints include the improvement in MIMVS scores assessed by blinded evaluators at 1 and 6 months after the last injection, as well as patient satisfaction.

General demographic data will be collected for both groups, including gender, age, BMI, and comorbidities. Basic vital signs such as blood pressure, heart rate, and body temperature before and after injections will also be recorded. The injection sites will be monitored for pain, tenderness, erythema, bruising, hematoma, or edema to assess the safety of the injectable product.

### 2.3 Statistical Analyses

The study defines three analysis populations: (1) full analysis set (FAS): includes all enrolled subjects who have used the device at least once, with exclusions made only in special circumstances; (2) per‐protocol set (PPS): a subset of the FAS, comprising subjects who received the specified treatment and have primary endpoint data; (3) safety set (SS): includes all enrolled subjects who have used the device at least once and have undergone safety assessments.

Statistical analysis will be performed using SAS 9.4. All statistical tests will be two‐sided, with a *p* value less than 0.05 considered to indicate statistical significance. Descriptive statistics for quantitative variables will include the number of cases, mean, standard deviation, median, minimum, and maximum values. For categorical variables, the description will include the number of cases and percentages. For intergroup comparisons, quantitative variables will be analyzed using either a *t*‐test for grouped data (based on data distribution) or the Wilcoxon rank‐sum test. Categorical variables will be analyzed using the chi‐square test or Fisher's exact test (if the chi‐square test is not applicable). Ordinal data will be analyzed using the Wilcoxon rank‐sum test or Cochran–Mantel–Haenszel (CMH) test.

## 3 Results

### 3.1 Subjects

**FIGURE 1:**
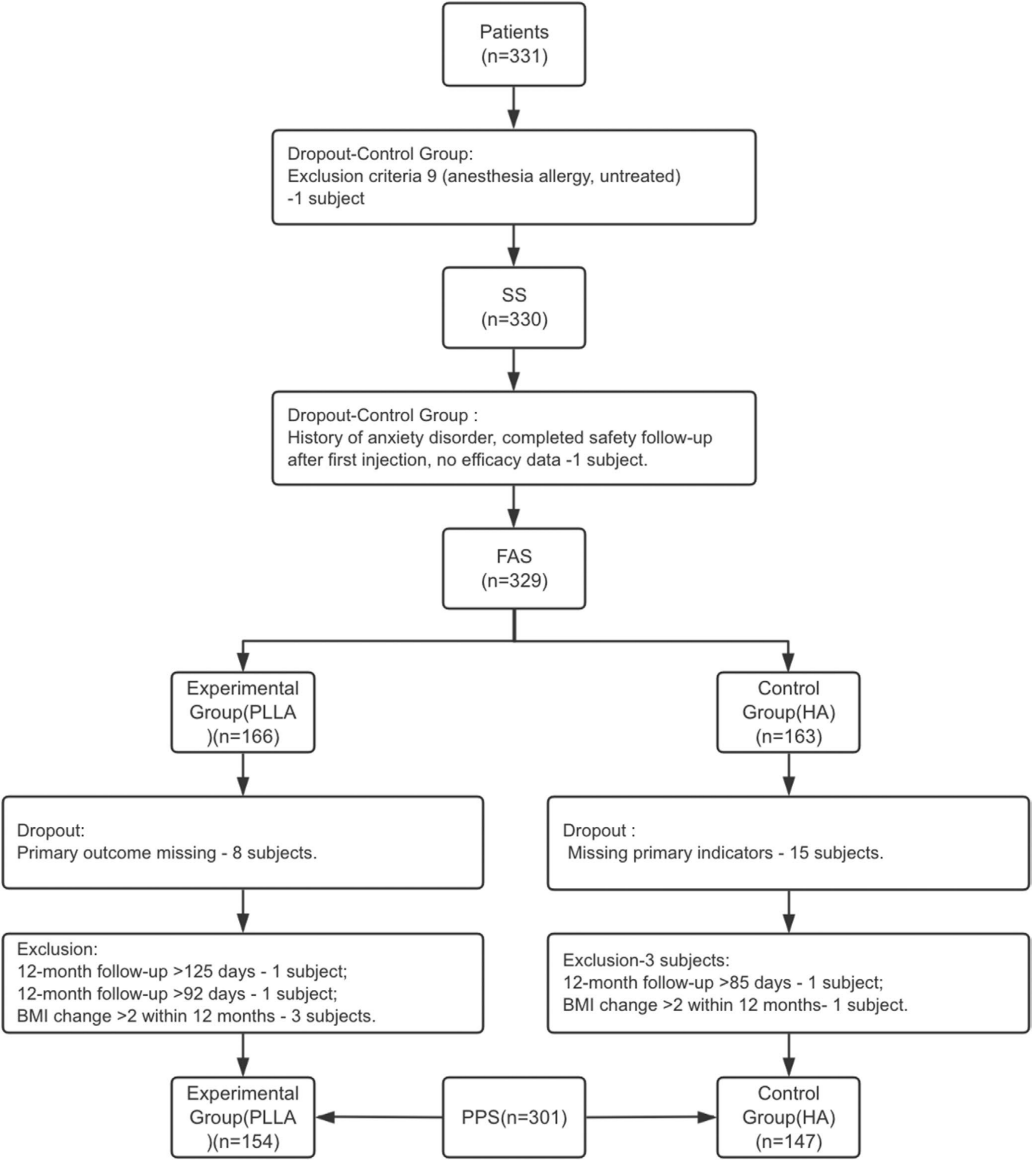
Subject disposition diagram.

A total of 331 subjects who met the inclusion criteria were enrolled between December 28, 2022 and August 28, 2024. Of these, 329 subjects were included in the full analysis set (FAS), with 166 subjects in the experimental group (injected with poly‐l‐lactic acid facial filler, SY3001‐2) and 163 subjects in the control group (injected with hyaluronic acid gel, Restylane Perlane Lidocaine). Among these, 23 subjects dropped out and seven were excluded. The per‐protocol set (PPS) included 301 subjects, with 154 in the experimental group and 147 in the control group. The safety set (SS) included 330 subjects, with 166 in the experimental group and 164 in the control group (Figure 1). The sample sizes for each analysis set met the statistical requirements outlined in the protocol.

Demographic and baseline characteristics were similar between the treatment groups (Table 1). Based on FAS analysis, the average age of the experimental group was 38.99 ± 9.10 years, whereas the control group had an average age of 40.01 ± 9.95 years (*p* > 0.05). In the experimental group, 9 subjects (5.42%) were male and 157 (94.58%) were female; in the control group, 9 subjects (5.52%) were male and 154 (94.48%) were female (*p* > 0.05). The average weight in the experimental group was 56.28 ± 8.15 kg, compared to 56.88 ± 9.09 kg in the control group (*p* > 0.05). The average height in the experimental group was 162.21 ± 5.98 cm, whereas in the control group it was 162.77 ± 6.27 cm (*p* > 0.05). The average BMI in the experimental group was 21.34 ± 2.51, compared to 21.42 ± 2.74 in the control group (*p* > 0.05).

**TABLE 1:** Demographic data.

Parameter	Indicator	Experimental group	Control group	Total	Statistic	*p*
Age (years)	*N* (missing)	166 (0)	163 (0)	329 (0)	0.535	0.593
	Mean (SD)	38.99 (9.10)	40.01 (9.95)	39.50 (9.53)	Wilcoxon rank‐sum test	
	Median	38.37	38.40	38.40		
	Q1, Q3	32.45, 43.61	33.81, 44.92	32.77, 44.22		
	Min, max	20.77, 69.26	23.94, 66.24	20.77, 69.26		
Gender	Male, *n* (%)	9 (5.42%)	9 (5.52%)	18 (5.47%)	0.002	0.968
	Female, *n* (%)	157 (94.58%)	154 (94.48%)	311 (94.53%)	Chi‐square test	
Weight (kg)	Mean (SD)	56.28 (8.15)	56.88 (9.09)	56.58 (8.62)	−0.097	0.992
	Median	56.00	55.00	55.50	Wilcoxon rank‐sum test	
	Q1, Q3	51.10, 60.00	51.00, 60.00	51.00, 60.00		
	Min, max	36.00, 89.50	40.00, 97.00	36.00, 97.00		
Height (cm)	Mean (SD)	162.21 (5.98)	162.77 (6.27)	162.48 (6.12)	0.786	0.432
	Median	162.00	162.00	162.00	Wilcoxon rank‐sum test	
	Q1, Q3	158.00, 166.00	159.00, 166.00	158.00, 166.00		
	Min, max	148.00, 182.00	147.00, 186.00	147.00, 186.00		
BMI (kg/m^2^)	Mean (SD)	21.34 (2.51)	21.42 (2.74)	21.38 (2.62)	−0.183	0.855
	Median	21.40	21.00	21.20	Wilcoxon rank‐sum test	
	Q1, Q3	19.70, 22.70	19.50, 22.80	19.60, 22.70		
	Min, max	14.60, 32.10	15.60, 33.20	14.60, 33.20		

*Note:* Age = (Date of Informed Consent Signing − Date of Birth + 1)/365.25.

Based on FAS analysis, 60 subjects (36.14%) in the experimental group had a history of medical conditions, whereas 59 subjects (36.20%) in the control group had a history of medical conditions. There was no statistically significant difference between groups (*p* > 0.05). In the experimental group, 1 subject (0.60%) had a history of allergies, compared to 5 subjects (3.07%) in the control group. Again, no statistically significant difference was found between the groups (*p* > 0.05) (Table 2).

**TABLE 2:** Past medical history.

Parameter	Indicator	Experimental group	Control group	Total	Statistic	*p*
Medical history (significant diseases/surgical history)	None, *n* (%)	106 (63.86%)	104 (63.80%)	210 (63.83%)	0.000	0.992
	Present, *n* (%)	60 (36.14%)	59 (36.20%)	119 (36.17%)	Chi‐square test	
Allergy history (including allergies to anesthetics, poly‐l‐lactic acid, hyaluronic acid sodium, or mannitol, etc.)	None, *n* (%)	165 (99.40%)	158 (96.93%)	323 (98.18%)	—	0.119
	Present, *n* (%)	1 (0.60%)	5 (3.07%)	6 (1.82%)	Fisher's exact test	

### 3.2 Effectiveness

**FIGURE 2:**
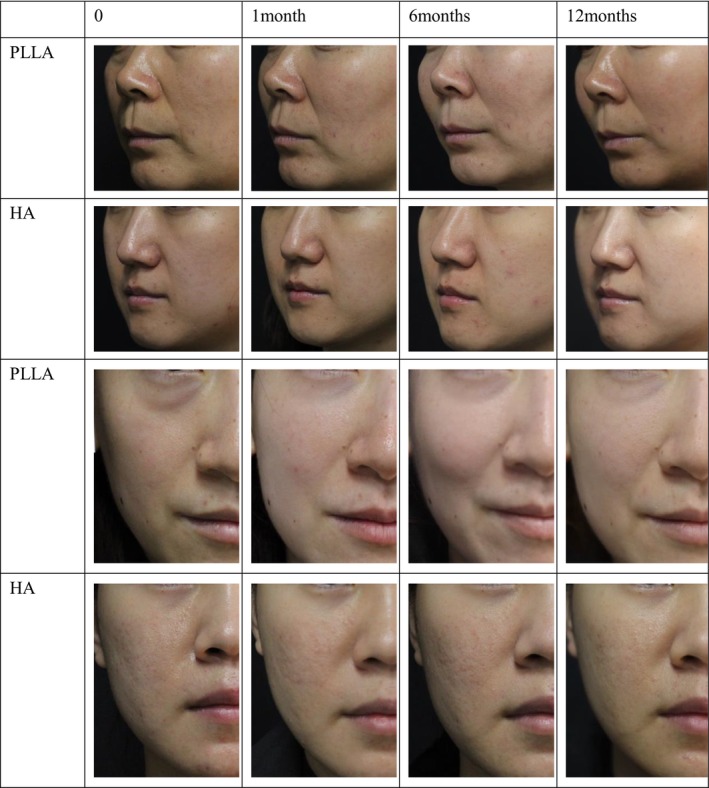
Changes in the middle part of the patient's face over time, treated with Poly‐l‐lactic acid (PLLA) (experimental group) and hyaluronic acid (HA) (control group). Images were taken at 1, 6, and 12 months posttreatment to show facial improvements. The patients' ages range from 30 to 40 years (top to bottom: 40, 37, 39, and 31 years). All patients are female.

Based on FAS and PPS analyses, the experimental group showed significantly higher MMVS score efficacy rates at 12 months after the last injection compared to the control group. The efficacy rates for FAS (no imputation), FAS (worst case imputation), and PPS were 90.57%, 86.75%, and 90.91%, respectively, whereas the control group had rates of 51.01%, 46.63%, and 51.02%, respectively. The 95% confidence intervals (CI) for the difference in efficacy between the two groups were both greater than 0%, indicating that the experimental group demonstrated superior efficacy compared to the control group (Table 3). Figure 2 shows photographs of the subjects before and after injection with PLLA and HA (at 1 month, 6 months, and 12 months).

**TABLE 3:** MMVS Score (blinded evaluator).

Parameter	Indicator	FAS		PPS	
		Experimental group	Control group	Experimental group	Control group
Screening phase left side	1 score, *n* (%)	0 (0.00%)	0 (0.00%)	0 (0.00%)	0 (0.00%)
	2 score, *n* (%)	69 (41.57%)	65 (39.88%)	67 (43.51%)	56 (38.10%)
	3 score, *n* (%)	86 (51.81%)	83 (50.92%)	76 (49.35%)	77 (52.38%)
	4 score, *n* (%)	11 (6.63%)	15 (9.20%)	11 (7.14%)	14 (9.52%)
	Total (missing)	166 (0)	163 (0)	154 (0)	147 (0)
	Statistic	0.394	CMH test	1.180	CMH test
	*p*	0.530		0.277	
Screening phase right side	1 score, *n* (%)	0 (0.00%)	0 (0.00%)	0 (0.00%)	0 (0.00%)
	2 score, *n* (%)	62 (37.35%)	67 (41.10%)	59 (38.31%)	57 (38.78%)
	3 score, *n* (%)	92 (55.42%)	80 (49.08%)	83 (53.90%)	76 (51.70%)
	4 score, *n* (%)	12 (7.23%)	16 (9.82%)	12 (7.79%)	14 (9.52%)
	Total (missing)	166 (0)	163 (0)	154 (0)	147 (0)
	Statistic	0.029	CMH test	0.032	CMH test
	*p*	0.864		0.859	
1‐Month post‐procedure left side	1 score, *n* (%)	88 (53.99%)	69 (44.81%)	82 (53.95%)	65 (44.52%)
	2 score, *n* (%)	68 (41.72%)	67 (43.51%)	63 (41.45%)	63 (43.15%)
	3 score, *n* (%)	7 (4.29%)	16 (10.39%)	7 (4.61%)	16 (10.96%)
	4 score, *n* (%)	0 (0.00%)	2 (1.30%)	0 (0.00%)	2 (1.37%)
	Total (missing)	163 (3)	154 (9)	152 (2)	146 (1)
	Statistic	5.936	CMH test	5.850	CMH test
	*p*	0.015		0.016	
1‐Month post‐procedure right side	1 score, *n* (%)	87 (53.37%)	75 (48.70%)	81 (53.29%)	71 (48.63%)
	2 score, *n* (%)	71 (43.56%)	63 (40.91%)	66 (43.42%)	59 (40.41%)
	3 score, *n* (%)	5 (3.07%)	14 (9.09%)	5 (3.29%)	14 (9.59%)
	4 score, *n* (%)	0 (0.00%)	2 (1.30%)	0 (0.00%)	2 (1.37%)
	Total (missing)	163 (3)	154 (9)	152 (2)	146 (1)
	Statistic	3.453	CMH test	3.370	CMH test
	*p*	0.063		0.066	
Left side decrease in grade	0‐grade level, *n* (%)	12 (7.36%)	18 (11.69%)	12 (7.89%)	18 (12.33%)
	1‐grade level, *n* (%)	118 (72.39%)	114 (74.03%)	111 (73.03%)	107 (73.29%)
	2‐grade level, *n* (%)	31 (19.02%)	21 (13.64%)	27 (17.76%)	20 (13.70%)
	3‐grade level, *n* (%)	2 (1.23%)	1 (0.65%)	2 (1.32%)	1 (0.68%)
	Total (missing)	163 (3)	154 (9)	152 (2)	146 (1)
	Statistic	3.227	CMH test	2.416	CMH test
	*p*	0.072		0.120	
Right side decrease in grade	0‐grade level, *n* (%)	7 (4.29%)	19 (12.34%)	6 (3.95%)	18 (12.33%)
	1‐grade level, *n* (%)	118 (72.39%)	106 (68.83%)	112 (73.68%)	101 (69.18%)
	2‐grade level, *n* (%)	37 (22.70%)	26 (16.88%)	33 (21.71%)	25 (17.12%)
	3‐grade level, *n* (%)	1 (0.61%)	3 (1.95%)	1 (0.66%)	2 (1.37%)
	Total (missing)	163 (3)	154 (9)	152 (2)	146 (1)
	Statistic	3.227	CMH test	2.416	CMH test
	*p*	0.072		0.120	
1‐Month post‐procedure efficacy	No, *n* (%)	13 (7.98%)	25 (16.23%)	12 (7.89%)	24 (16.44%)
	Yes, *n* (%)	150 (92.02%)	129 (83.77%)	140 (92.11%)	122 (83.56%)
	Total (missing)	163 (3)	154 (9)	152 (2)	146 (1)
	Statistic	5.119	Chi‐square test	5.118	Chi‐square test
	p	0.024		0.024	
6‐Month post‐procedure left side	1 score, *n* (%)	100 (63.29%)	58 (38.67%)	95 (63.33%)	55 (37.67%)
	2 score, *n* (%)	52 (32.91%)	65 (43.33%)	50 (33.33%)	64 (43.84%)
	3 score, *n* (%)	6 (3.80%)	24 (16.00%)	5 (3.33%)	24 (16.44%)
	4 score, *n* (%)	0 (0.00%)	3 (2.00%)	0 (0.00%)	3 (2.05%)
	Total (missing)	158 (8)	150 (13)	150 (4)	146 (1)
	Statistic	5.936	CMH test	5.850	CMH test
	*p*	0.015		0.016	
6‐Month post‐procedure right side	1 score, *n* (%)	94 (59.49%)	58 (38.67%)	89 (59.33%)	55 (37.67%)
	2 score, *n* (%)	59 (37.34%)	64 (42.67%)	56 (37.33%)	63 (43.15%)
	3 score, *n* (%)	5 (3.16%)	25 (16.67%)	5 (3.33%)	25 (17.12%)
	4 score, *n* (%)	0 (0.00%)	3 (2.00%)	0 (0.00%)	3 (2.05%)
	Total (missing)	158 (8)	150 (13)	150 (4)	146 (1)
	Statistic	23.089	CMH test	23.303	CMH test
	*p*	< 0.001		< 0.001	
Left side decrease in grade	−1‐grade level, *n* (%)	0 (0.00%)	1 (0.67%)	0 (0.00%)	1 (0.68%)
	0‐grade level, *n* (%)	9 (5.70%)	36 (24.00%)	8 (5.33%)	36 (24.66%)
	1‐grade level, *n* (%)	104 (65.82%)	93 (62.00%)	101 (67.33%)	90 (61.64%)
	2‐grade level, *n* (%)	42 (26.58%)	18 (12.00%)	38 (25.33%)	17 (11.64%)
	3‐grade level, *n* (%)	3 (1.90%)	2 (1.33%)	3 (2.00%)	2 (1.37%)
	Total (missing)	158 (8)	150 (13)	150 (4)	146 (1)
	Statistic	23.193	CMH test	22.770	CMH test
	*p*	< 0.001		< 0.001	
Right side decrease in grade	−1‐grade level, *n* (%)	0 (0.00%)	1 (0.67%)	0 (0.00%)	1 (0.68%)
	0‐grade level, *n* (%)	9 (5.70%)	42 (28.00%)	9 (6.00%)	42 (28.77%)
	1‐grade level, *n* (%)	102 (64.56%)	83 (55.33%)	97 (64.67%)	80 (54.79%)
	2‐grade level, *n* (%)	44 (27.85%)	21 (14.00%)	41 (27.33%)	21 (14.38%)
	3‐grade level, *n* (%)	3 (1.90%)	3 (2.00%)	3 (2.00%)	2 (1.37%)
	Total (missing)	158 (8)	150 (13)	150 (4)	146 (1)
	Statistic	23.103	CMH test	23.637	CMH test
	*p*	< 0.001		< 0.001	
6‐Month post‐procedure efficacy	No, *n* (%)	11 (6.96%)	46 (30.67%)	10 (6.67%)	46 (31.51%)
	Yes, *n* (%)	147 (93.04%)	104 (69.33%)	140 (93.33%)	100 (68.49%)
	Total (Missing)	158 (8)	150 (13)	150 (4)	146 (1)
	Statistic	28.669	Chi‐square test	29.761	Chi‐square test
	*p*	< 0.001		< 0.001	
12‐Month post‐procedure left side	1 score, *n* (%)	88 (55.35%)	29 (19.46%)	86 (55.84%)	29 (19.73%)
	2 score, *n* (%)	60 (37.74%)	80 (53.69%)	57 (37.01%)	78 (53.06%)
	3 score, *n* (%)	11 (6.92%)	36 (24.16%)	11 (7.14%)	24 (24.49%)
	4 score, *n* (%)	0 (0.00%)	4 (2.68%)	0 (0.00%)	4 (2.72%)
	Total (missing)	159 (7)	149 (14)	154 (0)	147 (0)
	Statistic	48.233	CMH test	47.151	CMH test
	*p*	< 0.001		< 0.001	
12‐Month post‐procedure right side	1 score, *n* (%)	83 (52.20%)	24 (16.11%)	81 (52.60%)	24 (16.33%)
	2 score, *n* (%)	67 (42.14%)	87 (58.39%)	64 (41.56%)	85 (57.82%)
	3 score, *n* (%)	9 (5.66%)	32 (21.48%)	9 (5.84%)	32 (21.77%)
	4 score, *n* (%)	0 (0.00%)	6 (4.03%)	0 (0.00%)	6 (4.08%)
	Total (missing)	159 (7)	149 (14)	154 (0)	147 (0)
	Statistic	52.241	CMH test	51.051	CMH test
	*p*	< 0.001		< 0.001	
Left side decrease in grade	−1‐grade level, *n* (%)	0 (0.00%)	1 (0.67%)	0 (0.00%)	1 (0.68%)
	0‐grade level, *n* (%)	15 (9.43%)	65 (43.62%)	14 (9.09%)	64 (43.54%)
	1‐grade level, *n* (%)	111 (69.81%)	74 (49.66%)	109 (70.78%)	73 (49.66%)
	2‐grade level, *n* (%)	31 (19.50%)	9 (6.04%)	29 (18.83%)	9 (6.12%)
	3‐grade level, *n* (%)	2 (1.26%)	0 (0.00%)	2 (1.30%)	0 (0.00%)
	Total (Missing)	159 (7)	149 (14)	154 (0)	147 (0)
	Statistic	49.204	CMH test	47.845	CMH test
	*p*	< 0.001		< 0.001	
Right side decrease in grade	−1‐grade level, *n* (%)	0 (0.00%)	1 (0.67%)	0 (0.00%)	1 (0.68%)
	0‐grade level, *n* (%)	10 (6.29%)	69 (46.31%)	9 (5.84%)	68 (46.26%)
	1‐grade level, *n* (%)	116 (72.96%)	72 (48.32%)	113 (73.38%)	71 (48.30%)
	2‐grade level, *n* (%)	31 (19.50%)	7 (4.70%)	30 (19.48%)	7 (4.76%)
	3‐grade level, *n* (%)	2 (1.26%)	0 (0.00%)	2 (1.30%)	0 (0.00%)
	Total (missing)	159 (7)	149 (14)	154 (0)	147 (0)
	Statistic	65.533	CMH test	65.001	CMH test
	*p*	< 0.001		< 0.001	
12‐Month post‐procedure efficacy	No, *n* (%)	15 (9.43%)	73 (48.99%)	14 (9.09%)	72 (48.98%)
	Yes, *n* (%)	144 (90.57%)	76 (51.01%)	140 (90.91%)	75 (51.02%)
	Total (missing)	159 (7)	149 (14)	154 (0)	147 (0)
	Statistic	57.987	CMH test	57.175	CMH test
	*p*	< 0.001		< 0.001	

**FIGURE 3:**
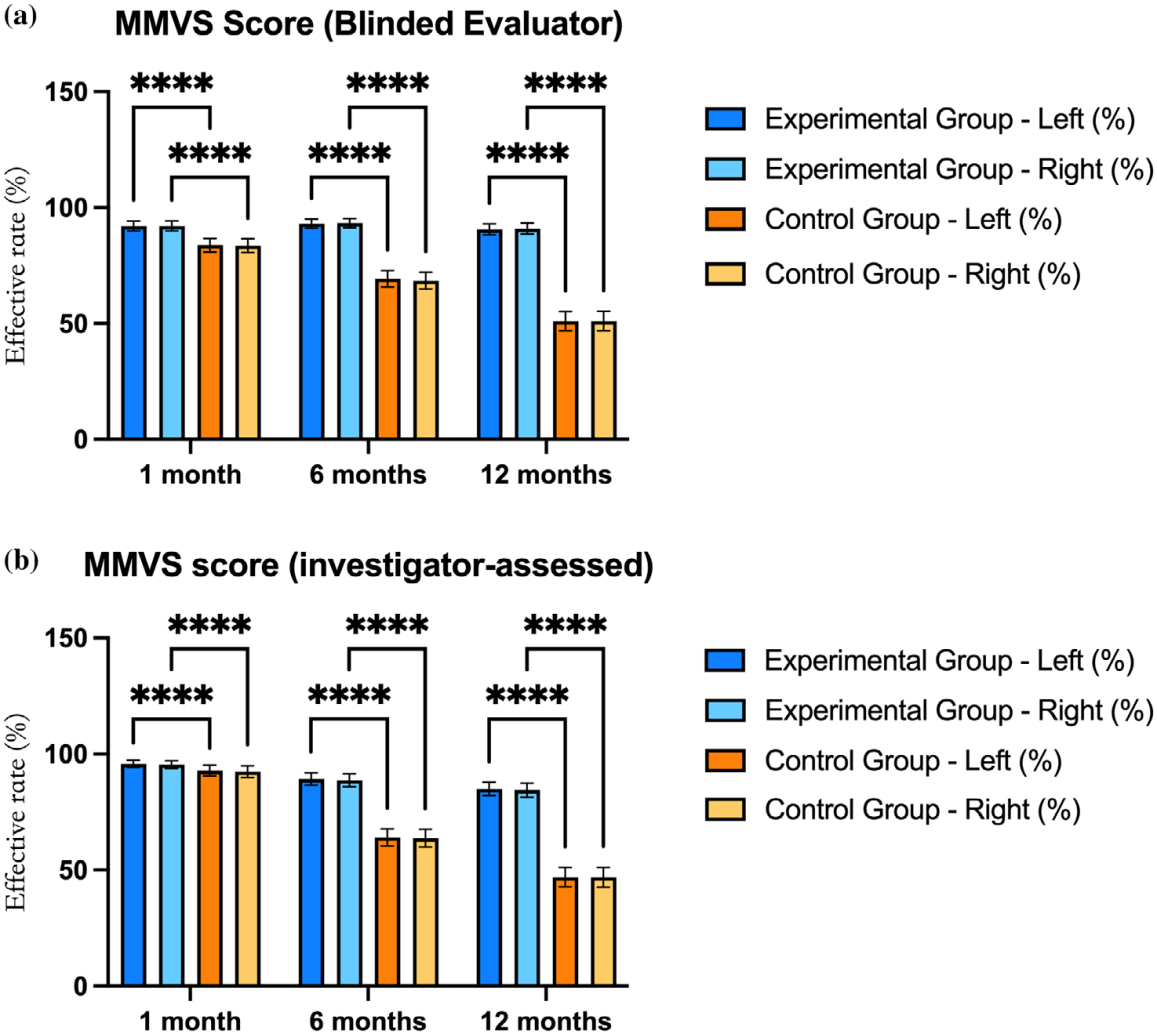
(a) MMVS score (blinded evaluator). (b) MMVS score (investigator‐assessed). The efficacy rates (%) of the experimental and control groups at 1 month, 6 months, and 12 months are shown. *p < 0.05; **p < 0.01; ***p < 0.001; ****P < 0.0001.

**FIGURE 4:**
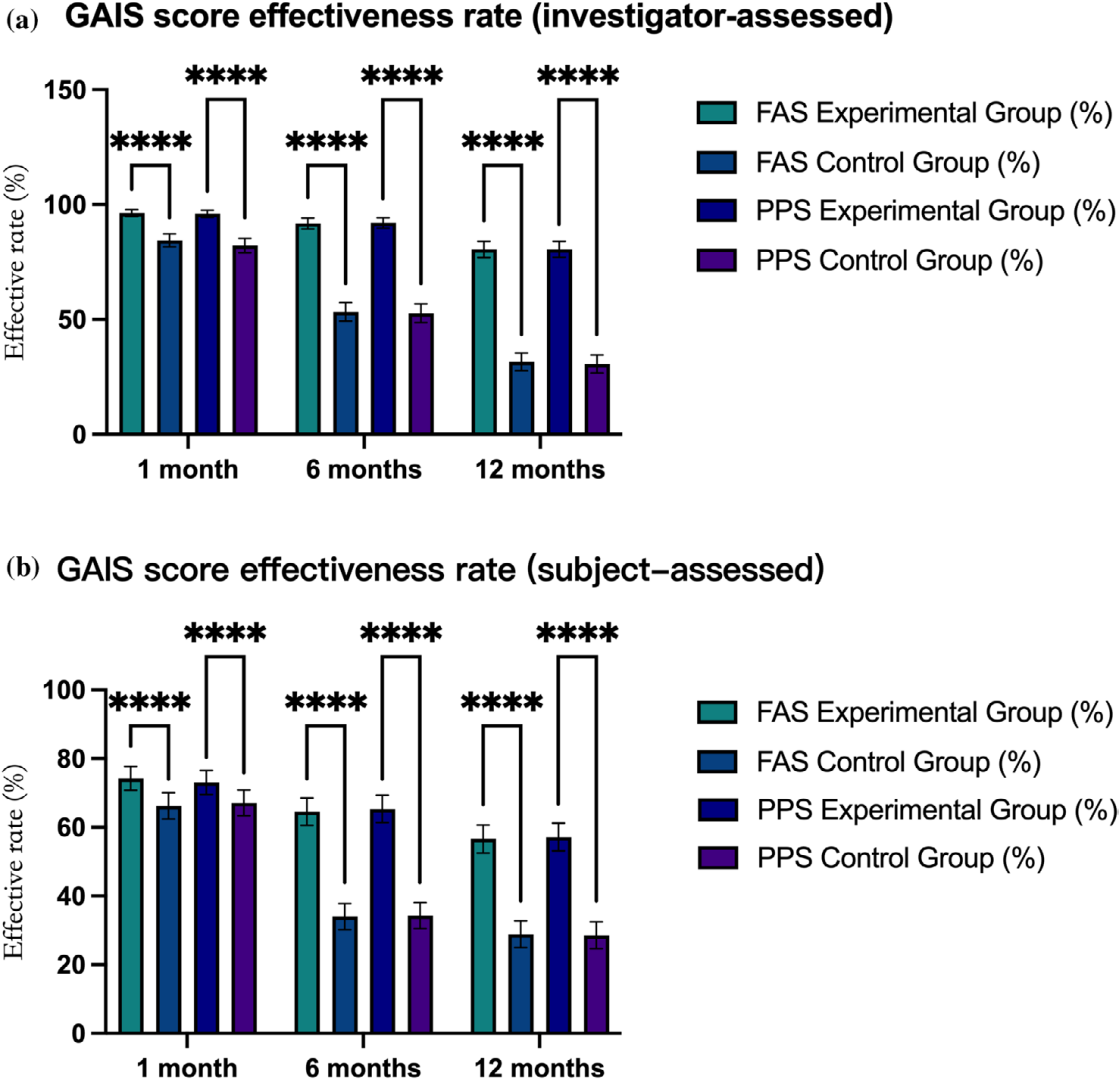
(a) GAIS score effectiveness rate (investigator‐assessed). (b) GAIS score effectiveness rate (subject‐assessed). The percentage of efficacy in the experimental and control groups, including both FAS and PPS populations, at 1 month, 6 months, and 12 months. *P < 0.05; **P < 0.01; ***P < 0.001; ****P < 0.0001.

Secondary endpoint analysis in this study revealed that for the MIMVS scores, the experimental group achieved an efficacy rate exceeding 92% at 1 month, significantly higher than the 83% in the control group (*p* < 0.05). At 6 months, the efficacy rate in the experimental group remained around 93%, whereas the control group decreased to approximately 69% (*p* < 0.05) (Table 3, Figure 3a). The investigator‐assessed results showed similar trends: at 1 month, there was no significant difference between the two groups (*p* > 0.05), but at 6 months, the efficacy rate in the experimental group remained close to 89%, whereas the control group dropped to 64% (*p* < 0.05). At 12 months, the efficacy rate in the experimental group maintained at 84%, whereas the control group further declined to 47% (*p* < 0.05) (Table 4, Figure 3b). Regarding GAIS scores, at 1 month, both groups had an efficacy rate of 100%. At 6 months, the experimental group maintained an efficacy rate of 99%, significantly higher than the control group's 86% (*p* ≤ 0.05). At 12 months, the efficacy rate in the experimental group was 94%, whereas the control group dropped to 74% (*p* < 0.05) (Table 5, Figure 4a). Similar results were found in the subject‐rated GAIS, with the experimental group showing significantly higher efficacy rates at both 6 and 12 months compared to the control group (*p* < 0.05) (Table 6, Figure 4b). In terms of subject satisfaction, at 6 months, the experimental group had a complete agreement rate of 60.13% for “treatment results met expectations” (compared to 52.00% in the control group, *p* < 0.05), and at 12 months, the “facial contour improvement” very satisfied rate was 45.91% in the experimental group (compared to 41.61% in the control group, *p* < 0.05) (Table 7). In summary, the experimental group outperformed the control group in MMVS and GAIS scores, as well as subject satisfaction, with the efficacy differences becoming more significant at 6 and 12 months.

**TABLE 4:** MMVS score (investigator‐assessed).

Parameter	Indicator	FAS		PPS	
		Experimental group	Control group	Experimental group	Control group
Screening phase left side	1 score, *n* (%)	0 (0.00%)	0 (0.00%)	0 (0.00%)	0 (0.00%)
	2 score, *n* (%)	71 (42.77%)	80 (49.08%)	68 (44.16%)	70 (47.62%)
	3 score, *n* (%)	66 (39.76%)	63 (38.65%)	57 (37.01%)	57 (38.78%)
	4 score, *n* (%)	29 (17.47%)	20 (12.27%)	29 (18.83%)	20 (13.61%)
	Total (missing)	166 (0)	163 (0)	154 (0)	147 (0)
	Statistic	2.122	CMH test	1.060	CMH test
	*p*	0.145		0.303	
Screening phase right side	1 score, *n* (%)	0 (0.00%)	0 (0.00%)	0 (0.00%)	0 (0.00%)
	2 score, *n* (%)	72 (43.37%)	85 (52.15%)	68 (44.16%)	75 (51.02%)
	3 score, *n* (%)	66 (39.76%)	59 (36.20%)	58 (37.66%)	54 (36.73%)
	4 score, *n* (%)	28 (16.87%)	19 (11.66%)	28 (18.18%)	18 (12.24%)
	Total (missing)	166 (0)	163 (0)	154 (0)	147 (0)
	Statistic	3.155	CMH test	2.344	CMH test
	*p*	0.076		0.126	
1‐Month post‐procedure left side	1 score, *n* (%)	94 (57.67%)	80 (51.95%)	88 (57.89%)	73 (50.00%)
	2 score, *n* (%)	65 (39.88%)	60 (38.96%)	60 (39.47%)	59 (40.41%)
	3 score, *n* (%)	4 (2.45%)	14 (9.09%)	7 (2.63%)	14 (9.59%)
	4 score, *n* (%)	0 (0.00%)	0 (0.00%)	0 (0.00%)	0 (0.00%)
	Total (missing)	163 (3)	154 (9)	152 (2)	146 (1)
	Statistic	3.316	CMH test	4.420	CMH test
	*p*	0.069		0.036	
1‐Month post‐procedure right side	1 score, *n* (%)	103 (63.19%)	90 (58.44%)	97 (63.82%)	82 (56.16%)
	2 score, *n* (%)	55 (33.74%)	50 (32.47%)	50 (32.89%)	50 (34.25%)
	3 score, *n* (%)	5 (3.07%)	14 (9.09%)	5 (3.29%)	14 (9.59%)
	4 score, *n* (%)	0 (0.00%)	0 (0.00%)	0 (0.00%)	0 (0.00%)
	Total (missing)	163 (3)	154 (9)	152 (2)	146 (1)
	Statistic	2.493	CMH test	3.840	CMH test
	*p*	0.114		0.050	
Left side decrease in grade	0‐grade level, *n* (%)	7 (4.29%)	8 (5.19%)	7 (4.61%)	8 (5.48%)
	1‐grade level, *n* (%)	103 (63.19%)	125 (81.17%)	96 (63.16%)	120 (82.19%)
	2‐grade level, *n* (%)	50 (30.67%)	21 (13.64%)	46 (30.26%)	18 (12.33%)
	3‐grade level, *n* (%)	3 (1.84%)	0 (0.00%)	3 (1.97%)	0 (0.00%)
	Total (missing)	163 (3)	154 (9)	152 (2)	146 (1)
	Statistic	13.620	CMH test	14.166	CMH test
	*p*	< 0.001		< 0.001	
Right side decrease in grade	0‐grade level, *n* (%)	2 (1.23%)	8 (5.19%)	2 (1.32%)	8 (5.48%)
	1‐grade level, *n* (%)	106 (65.03%)	122 (79.22%)	98 (64.47%)	118 (80.82%)
	2‐grade level, *n* (%)	53 (32.52%)	23 (14.94%)	50 (32.89%)	20 (13.70%)
	3‐grade level, *n* (%)	2 (1.23%)	1 (0.65%)	2 (1.32%)	0 (0.00%)
	Total (missing)	163 (3)	154 (9)	152 (2)	146 (1)
	Statistic	15.797	CMH test	20.122	CMH test
	*p*	< 0.001		< 0.001	
1‐Month post‐procedure efficacy	No, *n* (%)	7 (4.29%)	11 (7.14%)	7 (4.61%)	11 (7.53%)
	Yes, *n* (%)	156 (95.71%)	143 (92.86%)	145 (95.39%)	135 (92.47%)
	Total (missing)	163 (3)	154 (9)	152 (2)	146 (1)
	Statistic	1.200	Chi‐square test	1.126	Chi‐square test
	*p*	0.273		0.289	
6‐Month post‐procedure left side	1 score, *n* (%)	79 (50.00%)	51 (34.00%)	76 (50.67%)	50 (34.25%)
	2 score, *n* (%)	74 (46.84%)	66 (44.00%)	69 (46.00%)	63 (43.15%)
	3 score, *n* (%)	5 (3.16%)	27 (18.00%)	5 (3.33%)	27 (18.49%)
	4 score, *n* (%)	0 (0.00%)	6 (4.00%)	0 (0.00%)	6 (4.11%)
	Total (missing)	158 (8)	150 (13)	150 (4)	146 (1)
	Statistic	21.994	CMH test	21.704	CMH test
	*p*	< 0.001		< 0.001	
6‐Month post‐procedure right side	1 score, *n* (%)	88 (55.70%)	58 (38.67%)	85 (56.67%)	56 (38.36%)
	2 score, *n* (%)	64 (40.51%)	60 (40.00%)	59 (39.33%)	63 (39.73%)
	3 score, *n* (%)	6 (3.80%)	27 (18.00%)	6 (4.00%)	27 (18.49%)
	4 score, *n* (%)	0 (0.00%)	5 (3.33%)	0 (0.00%)	5 (3.42%)
	Total (missing)	158 (8)	150 (13)	150 (4)	146 (1)
	Statistic	20.619	CMH test	21.243	CMH test
	*p*	< 0.001		< 0.001	
Left side decrease in grade	−1‐grade level, *n* (%)	0 (0.00%)	1 (0.67%)	0 (0.00%)	1 (0.68%)
	0‐grade level, *n* (%)	12 (7.59%)	45 (30.00%)	12 (8.00%)	44 (30.14%)
	1‐grade level, *n* (%)	100 (63.29%)	97 (64.67%)	95 (63.33%)	95 (65.07%)
	2‐grade level, *n* (%)	44 (27.85%)	7 (4.67%)	41 (27.33%)	6 (4.11%)
	3‐grade level, *n* (%)	2 (1.27%)	0 (0.00%)	2 (1.33%)	0 (0.00%)
	Total (Missing)	158 (8)	150 (13)	150 (4)	146 (1)
	Statistic	48.162	CMH test	46.378	CMH test
	*p*	< 0.001		< 0.001	
Right side decrease in grade	−1‐grade level, *n* (%)	0 (0.00%)	1 (0.67%)	0 (0.00%)	1 (0.68%)
	0‐grade level, *n* (%)	10 (6.33%)	42 (28.00%)	10 (6.67%)	41 (28.08%)
	1‐grade level, *n* (%)	95 (60.13%)	100 (66.67%)	90 (60.00%)	99 (67.81%)
	2‐grade level, *n* (%)	52 (32.91%)	7 (4.67%)	49 (32.67%)	5 (3.42%)
	3‐grade level, *n* (%)	1 (0.63%)	0 (0.00%)	1 (0.67%)	0 (0.00%)
	Total (missing)	158 (8)	150 (13)	150 (4)	146 (1)
	Statistic	54.819	CMH test	55.012	CMH test
	*p*	< 0.001		< 0.001	
6‐Month post‐procedure efficacy	No, *n* (%)	17 (10.76%)	54 (36.00%)	17 (11.33%)	53 (36.30%)
	Yes, *n* (%)	141 (89.24%)	96 (64.00%)	133 (88.67%)	93 (63.70%)
	Total (missing)	158 (8)	150 (13)	150 (4)	146 (1)
	Statistic	27.637	Chi‐square test	25.545	Chi‐square test
	*p*	< 0.001		< 0.001	
12‐Month post‐procedure left side	1 score, *n* (%)	70 (44.03%)	24 (16.11%)	68 (44.16%)	23 (15.65%)
	2 score, *n* (%)	78 (49.06%)	90 (60.40%)	75 (48.70%)	89 (60.54%)
	3 score, *n* (%)	11 (6.92%)	30 (20.13%)	11 (7.14%)	30 (20.41%)
	4 score, *n* (%)	0 (0.00%)	5 (3.36%)	0 (0.00%)	5 (3.40%)
	Total (missing)	159 (7)	149 (14)	154 (0)	147 (0)
	Statistic	48.233	CMH test	36.298	CMH test
	*p*	< 0.001		< 0.001	
12‐Month post‐procedure right side	1 score, *n* (%)	78 (49.06%)	33 (22.15%)	76 (49.35%)	31 (21.09%)
	2 score, *n* (%)	71 (44.65%)	84 (56.38%)	68 (44.16%)	84 (57.14%)
	3 score, *n* (%)	10 (6.29%)	27 (18.12%)	10 (6.49%)	27 (18.37%)
	4 score, *n* (%)	0 (0.00%)	5 (3.36%)	0 (0.00%)	5 (3.40%)
	Total (missing)	159 (7)	149 (14)	154 (0)	147 (0)
	Statistic	31.534	CMH test	32.696	CMH test
	*p*	< 0.001		< 0.001	
Left side decrease in grade	−1‐grade level, *n* (%)	0 (0.00%)	1 (0.67%)	0 (0.00%)	1 (0.68%)
	0‐grade level, *n* (%)	20 (12.58%)	69 (46.31%)	20 (12.99%)	68 (46.26%)
	1‐grade level, *n* (%)	100 (62.89%)	75 (50.34%)	97 (62.99%)	75 (51.02%)
	2‐grade level, *n* (%)	38 (23.90%)	4 (2.68%)	36 (23.38%)	3 (2.04%)
	3‐grade level, *n* (%)	1 (0.63%)	0 (0.00%)	1 (0.65%)	0 (0.00%)
	Total (missing)	159 (7)	149 (14)	154 (0)	147 (0)
	Statistic	59.317	CMH test	58.252	CMH test
	*p*	< 0.001		< 0.001	
Right side decrease in grade	−1‐grade level, *n* (%)	0 (0.00%)	1 (0.67%)	0 (0.00%)	1 (0.68%)
	0‐grade level, *n* (%)	17 (10.69%)	65 (43.62%)	17 (11.04%)	65 (44.22%)
	1‐grade level, *n* (%)	98 (61.64%)	78 (52.35%)	95 (61.69%)	77 (52.38%)
	2‐grade level, *n* (%)	43 (27.04%)	5 (3.36%)	41 (26.62%)	4 (2.72%)
	3‐grade level, *n* (%)	1 (0.63%)	0 (0.00%)	1 (0.65%)	0 (0.00%)
	Total (missing)	159 (7)	149 (14)	154 (0)	147 (0)
	Statistic	61.690	CMH test	61.727	CMH test
	*p*	< 0.001		< 0.001	
12‐Month post‐procedure efficacy	No, *n* (%)	24 (15.09%)	79 (53.02%)	24 (15.58%)	78 (53.06%)
	Yes, *n* (%)	135 (84.91%)	70 (46.98%)	130 (84.42%)	69 (46.94%)
	Total (missing)	159 (7)	149 (14)	154 (0)	147 (0)
	Statistic	49.706	Chi‐square test	47.149	Chi‐square test
	*p*	< 0.001		< 0.001	

**TABLE 5:** GAIS score effectiveness rate (investigator‐assessed).

Parameter	Indicator	FAS		PPS	
		Experimental group	Control group	Experimental group	Control group
1‐month postoperative results	Very significant improvement, *n* (%)	49 (30.06%)	35 (22.73%)	45 (29.61%)	30 (20.55%)
	Significant improvement, *n* (%)	108 (66.26%)	95 (61.69%)	101 (29.61%)	30 (20.55%)
	Moderate improvement, *n* (%)	6 (3.68%)	24 (15.58%)	6 (3.95%)	24 (16.44%)
	No change, *n* (%)	0 (0.00%)	0 (0.00%)	0 (0.00%)	0 (0.00%)
	Worse than before, *n* (%)	0 (0.00%)	0 (0.00%)	0 (0.00%)	0 (0.00%)
	Total (missing)	163 (3)	154 (9)	152 (2)	146 (1)
	Statistic	8.836	CMH test	10.458	CMH test
	*p*	0.003		0.001	
6‐month postoperative results	Very significant improvement, *n* (%)	35 (22.15%)	11 (7.33%)	32 (21.33%)	11 (7.535%)
	Significant improvement, *n* (%)	110 (69.62%)	69 (46.00%)	107 (71.33%)	66 (45.21%)
	Moderate improvement, *n* (%)	12 (7.59%)	50 (33.33%)	10 (6.67%)	49 (33.56%)
	No change, *n* (%)	1 (0.63%)	20 (13.33%)	1 (0.67%)	20 (13.07%)
	Worse than before, *n* (%)	0 (0.00%)	0 (0.00%)	0 (0.00%)	0 (0.00%)
	Total (missing)	158 (8)	150 (13)	150 (4)	146 (1)
	Statistic	56.755	CMH test	55.699	CMH test
	*p*	< 0.001		< 0.001	
12‐month postoperative results	Very significant improvement, *n* (%)	34 (21.38%)	4 (2.68%)	32 (20.78%)	4 (2.72%)
	Significant improvement, *n* (%)	94 (59.12%)	43 (28.86%)	92 (59.74%)	41 (27.89%)
	Moderate improvement, *n* (%)	22 (13.84%)	64 (42.95%)	21 (13.64%)	64 (43.54%)
	No change, *n* (%)	9 (5.66%)	38 (25.50%)	9 (5.84%)	38 (25.85%)
	Worse than before, *n* (%)	0 (0.00%)	0 (0.00%)	0 (0.00%)	0 (0.00%)
	Total (missing)	159 (7)	149 (14)	154 (0)	147 (0)
	Statistic	73.338	CMH test	72.435	CMH test
	*p*	< 0.001		< 0.001	
1‐Month post‐procedure efficacy	No, *n* (%)	0 (0.00%)	0 (0.00%)	0 (0.00%)	0 (0.00%)
	Yes, *n* (%)	163 (100.00%)	154 (100.00%)	152 (100.00%)	146 (100.00%)
	Total (missing)	163 (3)	154 (9)	152 (2)	146 (1)
	Statistic	—		—	
	*p*	—		—	
6‐Month post‐procedure efficacy	No, *n* (%)	1 (0.63%)	20 (13.33%)	1 (0.67%)	20 (13.70%)
	Yes, *n* (%)	157 (99.37%)	130 (86.67%)	149 (99.33%)	126 (86.30%)
	Total (missing)	158 (8)	150 (13)	150 (4)	146 (1)
	Statistic	19.536	Chi‐square test	19.064	Chi‐square test
	*p*	< 0.001		< 0.001	
12‐Month post‐procedure efficacy	No, *n* (%)	9 (5.66%)	38 (25.50%)	9 (5.84%)	38 (25.85%)
	Yes, *n* (%)	150 (94.34%)	111 (74.50%)	145 (94.16%)	109 (74.15%)
	Total (missing)	159 (7)	149 (14)	154 (0)	147 (0)
	Statistic	23.421	Chi‐square test	22.846	Chi‐square test
	*p*	< 0.001		< 0.001	

**TABLE 6:** GAIS score effectiveness rate (subject‐assessed).

Parameter	Indicator	FAS		PPS	
		Experimental group	Control group	Experimental group	Control group
1‐month postoperative results	Very significant improvement, *n* (%)	63 (38.65%)	41 (26.62%)	57 (37.50%)	38 (26.03%)
	Significant improvement, *n* (%)	58 (35.58%)	61 (39.61%)	54 (35.53%)	60 (41.10%)
	Moderate improvement, *n* (%)	42 (25.77%)	52 (33.77%)	41 (26.97%)	48 (32.88%)
	No change, *n* (%)	0 (0.00%)	0 (0.00%)	0 (0.00%)	0 (0.00%)
	Worse than before, *n* (%)	0 (0.00%)	0 (0.00%)	0 (0.00%)	0 (0.00%)
	Total (missing)	163 (3)	154 (9)	152 (2)	146 (1)
	Statistic	5.077	CMH test	3.631	CMH test
	*p*	0.024		0.057	
6‐month postoperative results	Very significant improvement, *n* (%)	41 (25.95%)	20 (13.33%)	40 (26.67%)	19 (13.01%)
	Significant improvement, *n* (%)	61 (38.61%)	31 (20.67%)	58 (38.67%)	31 (21.23%)
	Moderate improvement, *n* (%)	54 (34.18%)	82 (54.67%)	51 (34.00%)	79 (54.11%)
	No change, *n* (%)	1 (0.63%)	17 (11.33%)	1 (0.67%)	17 (11.64%)
	Worse than before, *n* (%)	1 (0.63%)	0 (0.00%)	0 (0.00%)	0 (0.00%)
	Total (missing)	158 (8)	150 (13)	150 (4)	146 (1)
	Statistic	27.726	CMH test	30.477	CMH test
	*p*	< 0.001		< 0.001	
12‐month postoperative results	Very significant improvement, *n* (%)	34 (21.38%)	18 (12.08%)	34 (22.08%)	18 (12.24%)
	Significant improvement, *n* (%)	55 (34.59%)	25 (16.78%)	54 (35.06%)	24 (16.33%)
	Moderate improvement, *n* (%)	68 (42.77%)	63 (42.28%)	64 (41.56%)	62 (42.18%)
	No change, *n* (%)	2 (1.26%)	42 (28.19%)	2 (1.30%)	42 (28.57%)
	Worse than before, *n* (%)	0 (0.00%)	1 (0.67%)	0 (0.00%)	1 (0.68%)
	Total (missing)	159 (7)	149 (14)	154 (0)	147 (0)
	Statistic	36.045	CMH test	37.203	CMH test
	*p*	< 0.001		< 0.001	
1‐Month post‐procedure efficacy	No, *n* (%)	0 (0.00%)	0 (0.00%)	0 (0.00%)	0 (0.00%)
	Yes, *n* (%)	163 (100.00%)	154 (100.00%)	152 (100.00%)	146 (100.00%)
	Total (missing)	163 (3)	154 (9)	152 (2)	146 (1)
	Statistic	—		—	
	*p*	—		—	
6‐Month post‐procedure efficacy	No, *n* (%)	2 (1.27%)	17 (11.33%)	1 (0.67%)	17 (11.64%)
	Yes, *n* (%)	156 (98.73%)	133 (88.67%)	149 (99.33%)	129 (88.36%)
	Total (missing)	158 (8)	150 (13)	150 (4)	146 (1)
	Statistic	13.474	Chi‐square test	15.610	Chi‐square test
	*p*	< 0.001		< 0.001	
12‐Month post‐procedure efficacy	No, *n* (%)	2 (1.26%)	43 (28.86%)	2 (1.30%)	43 (29.25%)
	Yes, *n* (%)	157 (89.74%)	106 (71.14%)	152 (98.70%)	104 (70.75%)
	Total (missing)	159 (7)	149 (14)	154 (0)	147 (0)
	Statistic	46.970	Chi‐square test	46.218	Chi‐square test
	*p*	< 0.001		< 0.001	

**TABLE 7:** Injection site reaction severity grading within 4 weeks postinjection (SS).

Parameter	Indicator	Initial injection		First touch‐up injection		Second touch‐up injection
		Experimental group	Control group	Experimental group	Control group	SS
Pain	None, *n* (%)	67 (40.36%)	85 (51.83%)	83 (51.55%)	60 (68.18%)	76 (56.72%)
	Mild, *n* (%)	84 (50.60%)	70 (42.68%)	69 (42.86%)	24 (27.27%)	49 (36.57%)
	Moderate, *n* (%)	12 (7.23%)	9 (5.49%)	9 (5.59%)	2 (2.27%)	9 (6.72%)
	Severe, *n* (%)	3 (1.81%)	0 (0.00%)	0 (0.00%)	2 (2.27%)	0 (0.00%)
	Total	166 (0)	164 (0)	161 (0)	88 (0)	134 (0)
	Statistic	5.581	CMH test	3.479	CMH test	
	*p*	0.018		0.062		
Tenderness	None, *n* (%)	52 (31.33%)	76 (46.34%)	68 (42.24%)	60 (68.18%)	66 (49.25%)
	Mild, *n* (%)	78 (46.99%)	79 (48.17%)	75 (46.58%)	24 (27.27%)	54 (40.30%)
	Moderate, *n* (%)	32 (19.28%)	9 (5.49%)	16 (9.94%)	3 (3.41%)	13 (9.70%)
	Severe, *n* (%)	4 (2.41%)	0 (0.00%)	2 (1.24%)	1 (1.14%)	1 (0.75%)
	Total	166 (0)	164 (0)	161 (0)	88 (0)	134 (0)
	Statistic	18.476	CMH test	12.953	CMH test	
	*p*	< 0.001		< 0.001		
Local redness	None, *n* (%)	99 (59.64%)	111 (67.68%)	113 (70.19%)	73 (82.95%)	103 (76.87%)
	Mild, *n* (%)	54 (32.53%)	49 (29.88%)	42 (26.09%)	12 (13.64%)	27 (20.15%)
	Moderate, *n* (%)	12 (7.23%)	4 (2.44%)	6 (3.73%)	2 (2.27%)	4 (2.99%)
	Severe, *n* (%)	1 (0.60%)	0 (0.00%)	0 (0.00%)	1 (1.14%)	0 (0.00%)
	Total	166 (0)	164 (0)	161 (0)	88 (0)	134 (0)
	Statistic	4.522	CMH test	2.738	CMH test	
	*p*	0.033		0.098		
Bruising	None, *n* (%)	117 (70.48%)	105 (64.02%)	131 (81.37%)	72 (81.82%)	113 (84.33%)
	Mild, *n* (%)	39 (23.49%)	50 (30.49%)	28 (17.39%)	13 (14.77%)	20 (14.93%)
	Moderate, *n* (%)	8 (4.82%)	8 (4.88%)	2 (1.24%)	0 (0.00%)	1 (0.75%)
	Severe, *n* (%)	2 (1.20%)	1 (0.61%)	0 (0.00%)	3 (3.41%)	0 (0.00%)
	Total	166 (0)	164 (0)	161 (0)	88 (0)	134 (0)
	Statistic	0.598	CMH test	0.576	CMH test	
	*p*	0.439		0.448		
Hematoma	None, *n* (%)	149 (89.76%)	151 (92.07%)	148 (91.93%)	82 (93.18%)	119 (88.81%)
	Mild, *n* (%)	12 (7.23%)	9 (5.49%)	13 (8.07%)	3 (3.41%)	13 (9.70%)
	Moderate, *n* (%)	5 (3.01%)	4 (2.44%)	0 (0.00%)	3 (3.41%)	1 (0.75%)
	Severe, *n* (%)	0 (0.00%)	0 (0.00%)	0 (0.00%)	0 (0.00%)	1 (0.75%)
	Total	166 (0)	164 (0)	161 (0)	88 (0)	134 (0)
	Statistic	0.432	CMH test	0.251	CMH test	
	*p*	0.511		0.616		
Edema	None, *n* (%)	72 (43.37%)	127 (77.44%)	109 (67.70%)	81 (92.05%)	92 (68.66%)
	Mild, *n* (%)	67 (40.36%)	33 (20.12%)	40 (24.84%)	5 (5.68%)	28 (20.90%)
	Moderate, *n* (%)	25 (15.06%)	4 (2.44%)	11 (6.83%)	1 (1.14%)	12 (8.96%)
	Severe, *n* (%)	2 (1.20%)	0 (0.00%)	1 (0.62%)	1 (1.14%)	2 (1.49%)
	Total	166 (0)	164 (0)	161 (0)	88 (0)	134 (0)
	Statistic	42.901	CMH test	13.432	CMH test	
	*p*	< 0.001		< 0.001		
Other	None, *n* (%)	153 (92.17%)	155 (94.51%)	154 (95.65%)	86 (97.73%)	128 (95.52%)
	Mild, *n* (%)	9 (5.42%)	7 (4.27%)	6 (3.73%)	1 (1.14%)	5 (3.73%)
	Moderate, *n* (%)	2 (1.20%)	1 (0.61%)	1 (0.62%)	0 (0.00%)	1 (0.75%)
	Severe, *n* (%)	2 (1.20%)	1 (0.61%)	0 (0.00%)	1 (1.14%)	0 (0.00%)
	Total	166 (0)	164 (0)	161 (0)	88 (0)	134 (0)
	Statistic	0.888	CMH test	0.013	CMH test	
	*p*	0.346		0.909		

### 3.3 Safety

Based on the SS analysis, a total of 1703 adverse events were reported in 128 subjects in the experimental group, resulting in an incidence rate of 77.11%. In the control group, 875 adverse events occurred in 115 subjects, with an incidence rate of 70.12%. There was no statistically significant difference between the two groups (*p* > 0.05). Additionally, 459 subjects experienced 356 adverse events in total, yielding an incidence rate of 35.98%, with no significant difference between the groups (*p* > 0.05). In the experimental group, five patients experienced serious adverse events, including right lung cancer, acute enteritis, discogenic low back pain, mycoplasma pneumonia, and atypical endometrial hyperplasia. After a thorough evaluation, none of these adverse events were determined to be related to the study product. No serious adverse events were reported in the control group, and no significant difference was observed between the two groups (*p* > 0.05). Both groups did not experience any device‐related serious adverse events (Table 8). Regarding laboratory test results at screening, 1 month, and 12 months after the final injection, no significant abnormalities were observed in routine blood tests, urine tests, or liver and kidney function.

**TABLE 8:** Duration of injection site reactions within 4 weeks postinjection (SS).

Parameter	Indicator	Initial injection		First touch‐up injection		Second touch‐up injection
		Experimental group	Control group	Experimental group	Control group	SS
Pain	None, *n* (%)	67 (40.36%)	85 (51.83%)	83 (51.55%)	60 (68.18%)	76 (56.72%)
	1–3 days, *n* (%)	56 (33.73%)	58 (35.37%)	55 (34.16%)	20 (22.73%)	35 (26.12%)
	4–7 days, *n* (%)	30 (18.07%)	16 (9.76%)	14 (8.70%)	6 (6.82%)	16 (11.94%)
	8–14 days, *n* (%)	10 (6.02%)	2 (1.22%)	8 (4.97%)	2 (2.27%)	6 (4.48%)
	15–28 days, *n* (%)	3 (1.81%)	3 (1.83%)	1 (0.62%)	0 (0.00%)	1 (0.75%)
	Total	166 (0)	164 (0)	161 (0)	88 (0)	134 (0)
	Statistic	8.117	CMH test	5.450	CMH test	
	*p*	0.004		0.020		
Tenderness	None, *n* (%)	52 (31.33%)	76 (46.34%)	68 (42.24%)	60 (68.18%)	66 (49.25%)
	1–3 days, *n* (%)	29 (17.47%)	39 (23.78%)	51 (31.68%)	19 (21.59%)	26 (19.40%)
	4–7 days, *n* (%)	42 (25.30%)	29 (17.68%)	24 (14.91%)	6 (6.82%)	27 (20.15%)
	8–14 days, *n* (%)	35 (21.08%)	17 (10.37%)	13 (8.07%)	3 (3.41%)	12 (8.96%)
	15–28 days, *n* (%)	8 (4.82%)	3 (1.83%)	5 (3.11%)	0 (0.00%)	3 (2.24%)
	Total	166 (0)	164 (0)	161 (0)	88 (0)	134 (0)
	Statistic	15.692	CMH test	15.265	CMH test	
	*p*	< 0.001		< 0.001		
Local redness	None, *n* (%)	99 (59.64%)	111 (67.68%)	113 (70.19%)	73 (82.95%)	103 (76.87%)
	1–3 days, *n* (%)	49 (29.52%)	41 (25.00%)	34 (21.12%)	10 (11.36%)	24 (17.91%)
	4–7 days, *n* (%)	16 (9.64%)	6 (3.66%)	14 (8.70%)	3 (3.41%)	5 (3.73%)
	8–14 days, *n* (%)	2 (1.20%)	4 (2.44%)	0 (0.00%)	1 (1.14%)	2 (1.49%)
	15–28 days, *n* (%)	0 (0.00%)	2 (1.22%)	0 (0.00%)	1 (1.14%)	0 (0.00%)
	Total	166 (0)	164 (0)	161 (0)	88 (0)	134 (0)
	Statistic	0.908	CMH test	2.001	CMH test	
	*p*	0.341		0.157		
Bruising	None, *n* (%)	117 (70.48%)	105 (64.02%)	131 (81.37%)	72 (81.82%)	113 (84.33%)
	1–3 days, *n* (%)	21 (12.65%)	21 (12.80%)	16 (9.94%)	6 (6.82%)	11 (8.21%)
	4–7 days, *n* (%)	17 (10.24%)	21 (12.80%)	6 (3.73%)	5 (5.68%)	6 (4.48%)
	8–14 days, *n* (%)	9 (5.42%)	16 (9.76%)	6 (3.73%)	3 (3.41%)	3 (2.24%)
	15–28 days, *n* (%)	2 (1.20%)	1 (0.61%)	2 (1.24%)	2 (2.27%)	1 (0.75%)
	Total	166 (0)	164 (0)	161 (0)	88 (0)	134 (0)
	Statistic	2.021	CMH test	0.123	CMH test	
	*p*	0.155		0.726		
Hematoma	None, *n* (%)	149 (89.76%)	151 (92.07%)	148 (91.93%)	82 (93.18%)	119 (88.81%)
	1–3 days, *n* (%)	13 (7.83%)	5 (3.05%)	10 (6.21%)	4 (4.55%)	11 (8.21%)
	4–7 days, *n* (%)	4 (2.41%)	3 (1.83%)	3 (1.86%)	1 (1.14%)	2 (1.49%)
	8–14 days, *n* (%)	0 (0.00%)	4 (2.44%)	0 (0.00%)	1 (1.14%)	1 (0.75%)
	15–28 days, *n* (%)	0 (0.00%)	1 (0.61%)	0 (0.00%)	0 (0.00%)	1 (0.75%)
	Total	166 (0)	164 (0)	161 (0)	88 (0)	134 (0)
	Statistic	0.434	CMH test	0.003	CMH test	
	*p*	0.510		0.955		
Edema	None, *n* (%)	72 (43.37%)	127 (77.44%)	109 (67.70%)	81 (92.05%)	92 (68.66%)
	1–3 days, *n* (%)	42 (25.30%)	22 (13.41%)	25 (15.53%)	3 (3.41%)	18 (13.43%)
	4–7 days, *n* (%)	37 (22.29%)	9 (5.49%)	20 (12.42%)	2 (2.27%)	12 (8.96%)
	8–14 days, *n* (%)	11 (6.63%)	4 (2.44%)	3 (1.86%)	2 (2.27%)	9 (6.72%)
	15–28 days, *n* (%)	4 (2.41%)	2 (1.22%)	4 (2.48%)	0 (0.00%)	3 (2.24%)
	Total	166 (0)	164 (0)	161 (0)	88 (0)	134 (0)
	Statistic	42.901	CMH test	13.087	CMH test	
	*p*	< 0.001		< 0.001		
Other	None, *n* (%)	153 (92.17%)	155 (94.51%)	154 (95.65%)	86 (97.73%)	128 (95.52%)
	1–3 days, *n* (%)	6 (3.61%)	5 (3.05%)	1 (0.62%)	0 (0.00%)	1 (0.75%)
	4–7 days, *n* (%)	2 (1.20%)	3 (1.83%)	0 (0.00%)	0 (0.00%)	0 (0.00%)
	8–14 days, *n* (%)	1 (0.60%)	1 (0.61%)	2 (1.24%)	1 (1.14%)	1 (0.75%)
	15–28 days, *n* (%)	4 (2.41%)	0 (0.00%)	4 (2.48%)	1 (1.14%)	4 (2.99%)
	Total	166 (0)	164 (0)	161 (0)	88 (0)	134 (0)
	Statistic	2.017	CMH test	0.542	CMH test	
	*p*	0.156		0.462		

## 4 Discussion

This study, a prospective, multicenter, randomized, parallel‐controlled, assessor‐blinded superiority clinical trial, systematically evaluated the superiority of PLLA for correcting midface volume loss and contour defects. The results showed that the experimental group (PLLA) had a significantly higher efficacy rate than the control group (HA), with most subjects reporting high satisfaction with the outcomes. Compared to HA, PLLA not only significantly improved facial contour, but also provided more lasting cosmetic results by inducing endogenous collagen production. Additionally, the study observed potential benefits of PLLA in enhancing skin gloss and elasticity, which may be related to its regulation of adipocyte metabolism and anti‐inflammatory response mechanisms.

Poly‐l‐lactic acid (PLLA), a biodegradable synthetic polymer, has been widely used in tissue engineering and esthetic medicine due to its excellent biocompatibility and gradual degradation properties. Compared to traditional fillers like hyaluronic acid (HA), PLLA stimulates collagenogenesis to provide a gradual and lasting volume enhancement effect, which can last up to 2–3 years. The degradation of PLLA through hydrolysis generates lactic acid, which stimulates fibroblasts and macrophages, thereby inducing collagen synthesis and facilitating tissue augmentation. Moreover, PLLA has the potential to promote osteogenesis indirectly through its 3D scaffold structure, immunomodulation, degradation byproducts, and cell–cell interactions. Its osteogenic effect can be further enhanced by combining with functional materials [15, 16, 17]. However, uneven degradation rates, excessive local lactic acid concentrations, and foreign body reactions by macrophages may lead to fibrotic overgrowth and granuloma formation [18, 19, 20, 21]. Several studies have demonstrated that increasing the reconstitution volume and lowering the PLLA concentration can effectively reduce the risk of nodule formation. Palm et al. reported that Sculptra Aesthetic reconstituted to 8–10 mL resulted in a nodule rate of only 0.4% [22], significantly lower than the 6.9% observed at 5 mL [23]. Optimizing particle size, injection depth, and degradation kinetics may further reduce adverse events. In this study, the reconstitution concentration of PLLA was relatively high (49.2 mg/mL), and the incidence of nodules was 10.2% (17/166). Most nodules were mild (12 cases), whereas four cases were moderate or severe, all of which showed improvement over time. At follow‐up, 11 nodules had completely resolved without sequelae, and six cases showed improvement without serious complications. This may be related to the properties of the product used. SY3001‐2 features improved particle size uniformity, dispersibility, and purity, which may help reduce the risk of nodule formation even at higher concentrations. Nevertheless, it is necessary to further explore lower PLLA concentrations in future applications to reduce the incidence of complications.

In recent years, the mechanisms and clinical effects of PLLA and its optimized formulation (PLLA‐SCA) in facial rejuvenation have been confirmed through multidimensional studies. A 12‐month randomized controlled trial (NCT04124692) included 97 subjects in the treatment group and 52 in the control group. Using 3D photography, the results showed that the treatment group had a significantly greater improvement in Galderma cheek wrinkle score (GCWS) at 12 months compared to the control group (71.6% vs. 26.1%, *p* < 0.0001), confirming its clinical advantage in enhancing midface contour and reducing wrinkles [24]. Histological analysis further revealed that PLLA injection promoted collagen formation, angiogenesis, and tissue remodeling, with improved skin smoothness and gloss observed at 18 weeks, and 3D imaging confirmed reduced pore size and erythema [25]. Molecular mechanism studies showed that PLLA‐SCA activates adipocyte regeneration, metabolism, and collagen synthesis pathways while enhancing anti‐inflammatory responses and tissue remodeling, with a gene regulation pattern distinctly different from that of calcium hydroxylapatite (CaHA‐R), which primarily upregulates inflammatory genes like IL‐6. This modulation of adipocyte function not only improves skin elasticity and thickness but also optimizes the natural distribution and harmony of facial fat [26]. A study involving 25 healthy volunteers confirmed that absorbable suspension sutures (PLLA/PLGA) suspension sutures not only lifted tissues through cone fixation but also stimulated collagen regeneration, offering long‐term facial rejuvenation effects [27].

A systematic review compared four midface volumizing materials (PMMA, PLLA, CaHA, and autologous fat), highlighting the characteristics of each: PMMA (Bellafill) has a significant long‐term effect but requires skin testing; PLLA (Sculptra) offers gradual results with a sustainable effect for 2–3 years but has a risk of nodules; CaHA (Radiesse) combines immediate filling with collagen stimulation; and autologous fat has high safety but low volume retention (32% after 16 months). Despite high patient satisfaction, the studies are limited by sample size and follow‐up duration, requiring more long‐term data to validate durability [28]. In hard tissue repair, the performance differences of four generations of biodegradable materials (PLLA, PLLA/PGA, u‐HA/PLLA, and u‐HA/PLLA/PGA) were systematically assessed using a rat mandible model. The study found that first‐generation PLLA had limited osteogenesis and stronger inflammatory reactions, whereas second‐generation PLLA/PGA degraded faster and was suitable for non‐load‐bearing regions. Third‐generation u‐HA/PLLA and fourth‐generation u‐HA/PLLA/PGA demonstrated superior bioactivity and osteoconductivity, with the fourth‐generation material offering a shorter degradation time, making it an ideal choice for midface fixation. Key biomarkers (Runx2, OCN, CD68) supported the preclinical prospects of these novel materials in jaw and facial bone repair [29]. Additionally, the synergistic effects of treatment regimens were validated in multiple studies combining microneedling radiofrequency (MFR) with PLLA. Animal and clinical trials showed that MFR enhanced PLLA penetration via microchannels, significantly boosting its ability to stimulate collagen synthesis. Clinical data demonstrated that the combined treatment group outperformed the MFR‐alone group in terms of skin tightness and wrinkle improvement without adverse reactions. Researchers recommend combining MFR with PLLA for targeted delivery, improving efficacy and providing a safe and effective solution for skin laxity [30, 31, 32].

In this study, the efficacy rate of the PLLA group was significantly higher than that of the HA control group (90.57% vs. 51.01%), with continuous high safety and patient satisfaction during follow‐up. These results provide strong evidence for the wide clinical application of PLLA. First, PLLA‐SCA demonstrated significant efficacy in improving midface volume loss and contour defects, making it an ideal facial filler. Second, the study results indicated that PLLA‐SCA is highly safe, with mild and controllable adverse reactions, laying the foundation for its widespread clinical application. Finally, by optimizing adipocyte function and collagen generation, PLLA‐SCA offers a natural and lasting solution for facial rejuvenation. These findings not only expand the application of PLLA in the esthetic field but also offer new insights for personalized treatments.

## 5 Conclusion

In conclusion, the poly‐l‐lactic acid (PLLA) facial filler demonstrates significant efficacy in correcting facial volume loss and midfacial contour defects, with favorable safety profiles observed during long‐term use. Based on the full analysis set (FAS) and per‐protocol set (PPS), the effectiveness of the experimental group can be considered superior to that of the control group, indicating that the investigational device is effective. No serious adverse events related to the investigational device were observed in this clinical trial, and no device defects occurred, thus confirming the safety of the investigational device for clinical use. This study not only further validates the safety and efficacy of PLLA in the field of facial volumization but also highlights its unique advantages in improving facial structure and volume. It provides new insights for the development of personalized and precise esthetic treatment protocols in the future.

## Author Contributions

Y.Z. wrote the manuscript and created the figures. X.Z. and H.Z. designed the experiments. X.G. and Y.W. analyzed the data. W.Q., Z.S., J.D., S.B., and R.R. performed the experiments. All authors have read and approved the final manuscript.

## Ethics Statement

The clinical trial protocol (V1.1, July 28, 2022) was approved by the Beijing Hospital Ethics Committee on September 19, 2022, approval number: 2022 BJYYEC‐187. The protocol was approved by the Sichuan University West China Hospital Ethics Committee on December 8, 2022, approval number: WCHSIRB‐D‐2022‐489. The protocol was approved by the Ethics Committee of Hangzhou First People's Hospital on October 27, 2022, approval number: 2022‐YLS‐No. 086. The protocol was approved by the Ethics Committee of the Chinese Academy of Medical Sciences Peking Union Medical College Hospital on December 15, 2022, approval number: 2022‐LS‐No. 044.

## Conflicts of Interest

The authors declare no conflicts of interest.

## Data Availability

The data underlying this article will be shared on reasonable request to the corresponding author.
